# A DNA barcode library for the water mites of Montenegro

**DOI:** 10.3897/BDJ.9.e78311

**Published:** 2021-12-20

**Authors:** Vladimir Pešić, Andrzej Zawal, Ana Manović, Aleksandra Bańkowska, Milica Jovanović

**Affiliations:** 1 Department of Biology, University of Montenegro, Podgorica, Montenegro Department of Biology, University of Montenegro Podgorica Montenegro; 2 Institute of Marine and Environmental Sciences, Center of Molecular Biology and Biotechnology, University of Szczecin, Szczecin, Poland Institute of Marine and Environmental Sciences, Center of Molecular Biology and Biotechnology, University of Szczecin Szczecin Poland; 3 Institute of Biology, University of Szczecin, Szczecin, Poland Institute of Biology, University of Szczecin Szczecin Poland

**Keywords:** DNA barcoding, COI, water mites, Montenegro, species delimitation

## Abstract

Water mites (Acari, Hydrachnidia) are a significant component of freshwater ecosystems inhabiting a wide range of aquatic habitats. This study provides a first comprehensive DNA barcode library for the water mites of Montenegro. DNA barcodes were analysed from 233 specimens of water mites morphologically assigned to 86 species from 28 genera and 15 families. In the course of the study, four species, i.e. *Lebertiareticulata* (Koenike, 1919), *Atractidesinflatipalpis* K.Viets, 1950, *A.latipes* (Szalay, 1935) and *Parabrachypodamontii* (Maglio, 1924) were molecularly confirmed as new for Montenegro and three species, i.e. *Protziaoctopora* Lundblad, 1954, *Pionalaminata* (Thor, 1901) and *Unionicolaypsilophora* (Bonz, 1783) are new for the Balkan Peninsula. Results are analysed using the Barcode Index Number system (BIN) and the Refined Single Linkage (RESL) of BOLD. The BIN assigned sequences to 98 clusters, while the RESL reveal 103 operational taxonomic units (OTUs). Unique BINs were revealed for 72 species (83.7%), whereas twelve species (14%) were characterised by two BINs and two species (2.3%) with three BINs. Amongst the studied taxa, 14 species were found with a high intraspecific sequence divergences (˃ 2.2%), emphasising the need for additional comprehensive morphological and molecu­lar analysis of these species.

## Introduction

Hydrachnidia, also known as water mites, is a most diverse and abundant group of arachnids in freshwater habitats (Davids et al. 2007). With nearly 7,500 species grouped into 550 genera (Smit 2020), they inhabit a wide range of aquatic habitats, including lotic, lentic, interstitial and temporary waters. Water mites have a complex life cycle that includes two pupa-like resting stages, i.e. protonymph and tritonymph and three active stages: larva is almost always parasitic, deutonymphs and adults that are predators of minute invertebrates (Davids et al. 2007). Some recent studies have shown that water mites can be good indicators of ecosystem health, especially of groundwater-dependent ecosystems (Pešić et al. 2019b). However, their time-consuming taxonomic identification has been identified as a major constraint for more significant involvement in rapid assessment programmes (Weigand et al. 2019).

Traditional morphology often underestimates the true diversity of water mites and, in recent years, it has been successfully replaced by an integrative approach that combines both morphological characteristics and molecular data (Martin et al. 2010, Pešić et al. 2017, Fisher et al. 2017, Pešić et al. 2019a, Pešić et al. 2020d, Pešić and Smit 2020). This process has been enhanced by the formation of the comprehensive DNA barcode reference libraries, such as the BOLD System (https://www.boldsystems.org/) and GenBank (https://www.ncbi.nlm.nih.gov/). DNA barcodes have been proposed and successfully adopted for water mites as an efficient method for detecting previously overlooked and/or misidentified species (Martin et al. 2010, Pešić et al. 2017, Pešić et al. 2019). The significant increase in the number of studies using DNA barcodes in recent years, especially in some regions, has laid the foundations for building a comprehensive library of DNA barcodes at the national and/or regional level (e.g. Blattner et al. 2019).

Of the Balkan countries, Montenegro is one of the best studied from the taxonomic and faunistic point of view (Pešić et al. 2018). Water mite research began in 1903 when the Czech zoologist Karl Thon published the first list of 13 species (Thon 1903). For more than one century, a large number of papers on the Montenegrin water mites have been published (Musselius 1912, Viets 1936, Pešić 2001, Pešić 2002b, Pešić 2002d, Pešić 2002a, Pešić 2002c, Pešić 2003a, Pešić 2003c, Pešić and Gerecke 2003, Di Sabatino et al. 2003, Pešić 2003b, Pešić 2004b, Pešić 2004a, Smit and Pešić 2004, Baker et al. 2008, Pešić et al. 2010, Pešić et al. 2012, Pešić et al. 2017, Bańkowska et al. 2016, Pešić et al. 2018, Zawal and Pešić 2018, Pešić et al. 2019a, Pešić et al. 2019c, Pešić et al. 2020a, Pešić et al. 2020b, Pešić et al. 2020c, Pešić et al. 2020d, Zawal et al. 2020, Pešić and Smit 2020, Pešić et al. 2021b)

Currently, 201 species of water mites have been reported for Montenegro (Pešić et al. 2018, Pešić et al. 2019c, Pešić et al. 2020c, Pešić et al. 2020d, Pešić and Smit 2020). This number makes up about 50% of the species known from the Balkans, which is estimated at about 400 species (Pešić et al. 2018). This is still a small number for the area of such hydrogeological characteristics and the turbulent geological history as the Balkans. Therefore, there is no reason not to believe that the expected number of water mites in the Balkans is at least at the level of Central Europe which is home to approximately 745 species (Gerecke et al. 2016).

The aim of the study is to develop and evaluate the first library of barcodes for water mites from Montenegro, targeting a COI fragment of ~ 658 bp. Taking advantage of publicly available DNA barcode reference libraries, such as the BOLD and the use of the universal Barcode Index Number (BIN), allows us to assess the molecular diversity of water mite species inhabiting the territory of Montenegro, as well as to explore their distribution patterns in Europe. Moreover, this approach will allow us to also identify problematic species groups both for traditional taxonomy and for DNA barcoding.

## Material and methods

**Figure 1.**
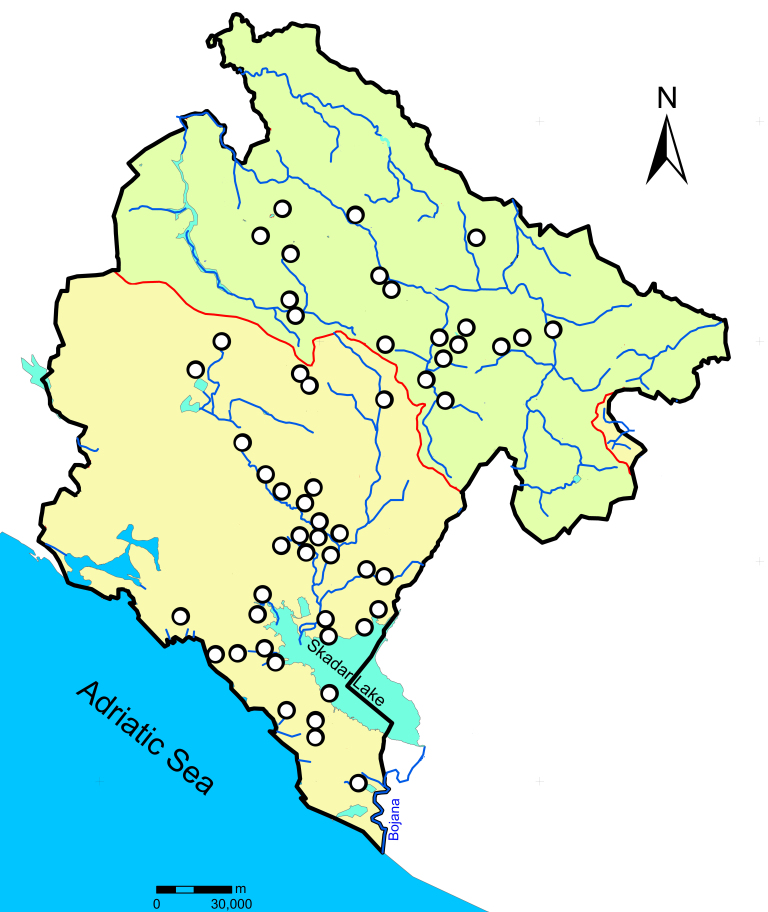
Sampling sites from Montenegro. The green colour represents the Danube Basin (Black Sea) and the yellow colour represents the Adriatic Basin.

Water mites were collected by hand netting, sorted live in the field and immediately preserved in 96% ethanol (EtOH) for the molecular analysis. Water mites were collected from 54 sampling sites in Montenegro (Fig. 1) during several sampling campaigns from 2018-2020. Photos from each studied specimen were taken before mo­lecular work started. The photographs were made using a camera on a Samsung Galaxy smartphone.

Molecular analysis were conducted in the Canadian Centre for DNA Barcoding (Guelph, Ontario, Canada; (CCDB; http://ccdb.ca/) and in the Department of Invertebrate Zoology and Hydrobiology (DIZH), University of Łódź, Poland. For the methods used for cytochrome *c* oxidase subunit I (COI) gene amplification in DIZH, see Pešić et al. (2017). In CCDB, the specimens were sequenced for the barcode region of COI using standard invertebrate DNA extraction (Ivanova et al. 2007), amplification (Ivanova and Grainger 2007a) and sequencing protocols (Ivanova and Grainger 2007b). The DNA extracts were archived in −80°C freezers at the Centre for Biodiversity Genomics (CBG; biodiversitygenomics.net) and the specimen vouchers were stored in 95% EtOH and returned to the first author for morphological examination. Some of these vouchers were dissected as described elsewhere (Davids et al. 2007) and slide-mounted in Faure’s medium, while the rest were transferred to Koenike’s medium and stored in the collection of the first author at the Department of Biology in Podgorica.

### DNA barcode analysis

In CCDB, the chromatograms were assembled into consensus sequences for each specimen and uploaded to BOLD. The taxonomic account, voucher specimen ID, collecting locality and voucher depositor were incorporated into the system for further analysis. Water mite sequences, obtained during this study, were grouped in the “MNHYD” (DNA barcode reference library of Montenegrin water mites) dataset. Detailed voucher information, taxonomic classifications, photos, DNA barcode sequences, primer pairs used and trace files (including their quality) were uploaded to the dataset “MNHYD” on the Barcode of Life Data Systems (BOLD; www.boldsystems.org).

The translation of the *COI* sequences into amino acids did not contain any stop codon positions and blasting the sequences confirmed the absence of contaminations. In cases of the four *Unionicolaypsylophora* mites, we amplified *Anodontaexulcerata* DNA instead of water mite DNA. These specimens were excluded from further analysis.

The reference library for the molecular identification of water mites sequenced in this study was analysed using the Barcode Index Number system (BIN) (Felsenstein 1985). The distribution of BINs was performed by the Barcode of Life Data System v.4 (accessed 15 November 2021). The two-phase BIN analysis system in the first phase applies a first threshold of 2.2% (that allows a rough differentiation between intraspecific and interspecific distances), followed by refinements through Markov clustering into the final BINs (Ratnasingham and Hebert 2013). BOLD ID and accession numbers for all specimens included in final dataset are given in Table 1.

All obtained BINs were inspected for concordance using BOLD Workbench. The Refined Single Linkage (RESL) algorithm was used to assign water mite barcodes to Operational Taxonomic Units (OTUs).

Sequence comparisons were performed using MUSCLE alignment (Edgar 2004). Intra- and interspecific genetic distances were calculated, based on the Kimura 2-parameter model (K2P; Kimura 1980), using MEGA-X, version 10.1 (Kumar et al. 2018). The Neighbour-Joining (NJ) tree (edited in MEGA7, Kumar et al. 2016), based on K2P distances and pairwise deletion of missing data, was used to visualise similarity. The support for tree branches was calculated by the non-parametric bootstrap method (Felsenstein 1985) with 1000 replicates and shown next to the branches.

## Results

DNA barcodes of 233 specimens morphologically assigned to 86 species from 28 genera and 15 families of water mites from Montenegro were newly generated for this study. The specimens were collected through the “DNA-Eco” (DNA barcode reference library as a tool for sustainable management of freshwater ecosystems in the highly threatened Lake Skadar Basin) project. The current study develops the first COI barcode reference library of water mites for Montenegro with the focus on Skadar/Shkodra Lake catchment area.

Fragment lengths of the analysed DNA barcode fragments ranged from 201 to 658 (mean: 636.2) base pairs, including no stop codons, insertions or deletions. The DNA barcode re­gion was characterised by a high AT-content: the mean sequence compositions were A = 30.82 ± 0.1252%, C = 20.39 ± 0.1222%, G = 14.91 ± 0.0709% and T = 33.88 ± 0.1253%. The obtained results are similar to those found in other arthropod studies (e.g. Raupach et al. 2015).

The families Hygrobatidae Koch, 1842 and Lebertiidae Thor, 1900 are represented by the highest number of sequences (53 and 44, respectively). The opposite, the three families Arrenuridae Thor, 1900, Teutoniidae Koenike, 1910 and Limnesiidae Thor, 1900 are represented each with two sequences and the two familes Athienemanniidae K. Viets, 1922 and Wettinidae Cook, 1956 by the lowest number of sequences (each with one sequence). The most common genus was *Lebertia* Neuman, 1880, for which 44 barcode sequences (11 species) were generated, followed by *Atractides* Koch, 1837 (35 barcodes; 13 species), *Torrenticola* Piersig, 1896 and *Sperchon* Kramer, 1877 (29 and 26 barcodes, 10 and 8 species, respectively). Six genera were represented by a single specimen. The highest number of barcodes per species was reached for *Atractidespennatus* (K. Viets, 1922), *Sperchonviolaceus* Walter, 1944 and *Torrenticolameridionalis* Di Sabatino and Cicolani, 1990 (each with 10 barcodes), followed by *Lebertiainaequalis* (Koch, 1837) and *L.variolata* Gerecke, 2009 (each with 8 barcodes) and *Sperchonthienemanni* Koenike, 1907 (6 barcodes). On the other hand, most species are represented by less than 5 DNA barcodes. Thirty-three species are represented by a single DNA barcode not allowing us to estimate the intraspecific distances. BOLD ID and accession numbers for all specimens included in final dataset are given in Table 1.

The mean intrageneric K2P distance was 20.2 ± 0.0% (range 6.09-42.37%). The mean intraspecific nucleotide K2P distances were 2.43 ± 0.01% (ranging from 0% to 24.16%). The summary statistics showing significant changes of average K2P distances within the different taxonomic levels are given in Table 2.

The BIN and RESL (OTU) analyses assigned sequences to 98 BINs and 103 OTUs, respectively. Fifty BINs (159 records) were concordant (51%) and 48 BINs were represented by a single sequence (49%). At the time of publication of the dataset, fifty-five (56.1%) of these BINs (with 102 sequences) included sequences only from Montenegro, while the remaining BINs included sequences also from other countries.

Most of the morphologically-identified species show an intraspecific variation of less than 2%. However, the 14 taxa listed in Table 3 showed a maximum interspecific divergence larger than 2%, resulting in these species in BOLD being spread over more than one BIN. Two species, *Lebertiaglabra* Thor, 1897 and *L.inaequalis* appeared each with 3 BINs and twelve species, i.e. *Lebertiamaculosa* Koenike, 1902, *L.porosa* Thor, 1900, *Sperchonbrevirostris* Koenike, 1895, *S.clupeifer* Piersig, 1896, *Sperchonopsisverrucosa* (Protz, 1896), *Monatractidesmadritensis* (K. Viets, 1930), *Torrenticolameridionalis, T. laskai* Di Sabatino, 2009, *Atractidesgibberipalpis* Piersig, 1898, *A.nodipalpis* Thor, 1899, *Hygrobatescalliger* Piersig, 1896 and *Unionicolaminor* (Soar, 1900), each with 2 BINs (Table 3). In total, unique BINs were revealed for 72 species (83.7%), two BINs for 12 species (14.0%) and three BINs for two species (2.3%).

The NJ analyses, based on K2P distances, revealed non-overlapping clusters with bootstrap support values > 95% for 50 species (58%) with more than one analysed specimen indicating a high congruence between BINs affiliation and morphological species identification. Moreover, specimens showing high intraspecific distances are also clearly separated into different clades. A more detailed topology of all analysed specimens is presented in the supporting information (Suppl. material 2).

## Discussion

This study provides COI barcodes for 233 specimens representing 86 morphologically identified species of water mites from Montenegro. These represent 42.8% of Montenegrin water mite fauna, based on Pešić et al. (2018) and papers published thereafter (Pešić et al. 2019c, Pešić et al. 2020a, Pešić et al. 2020c, Pešić et al. 2020d, Pešić and Smit 2020). BOLD and RESL (OTU) analyses revealed 98 BINs and 103 OTUs, respectively, highlighting the high molecular diversity of the water mite fauna of Montenegro.

Of the 86 species recorded in this study, 79 species were previously reported for Montenegro. DNA barcoding confirmed the presence of four species new for Montenegro, i.e. *Lebertiareticulata* (Koenike, 1919), *Atractidesinflatipalpis* K.Viets, 1950, *A.latipes* (Szalay, 1935) and *Parabrachypodamontii* (Maglio, 1924). Three species, i.e. *Protziaoctopora* Lundblad, 154, *Pionalaminata* (Thor, 1901) and *Unionicolaypsilophora* (Bonz, 1783) are recorded for the first time for the Balkan Peninsula. Specimens of the latter species were found between the gill blades of mussels *Anodontaexulcerata* Clesin, 1876, whose identification was confirmed by molecular data.

Moreover, species identification, based on molecular data conducted during this project, extended the list of Montenegrin water mites by description of several species new for science, i.e. *Atractidesanae* Pešić, 2020, *Hygrobateslacrima* Pešić, 2020, *H.limnocrenicus* Pešić, 2020, *H mediterraneus* Pešić, 2020 and *Mideopsismilankovici* Pešić and Smit, 2020 (Pešić et al. 2020a, Pešić et al. 2020c, Pešić et al. 2020d, Pešić and Smit 2020). All of these studies highlighted the importance of an integrated approach that combines the morphology-based taxonomy and DNA barcodes.

Our study confirmed efficiency of DNA barcoding as a tool for the identification of water mites. In particular, 72 of the 86 morphologically-identified species exactly matched the BINs defined from BOLD. This result coincides with high identification efficiency rates through the BOLD *Best Close Match* analysis. Nevertheless, our data revealed also 14 species listed in Table 3 that showed high intraspecific distances (> 2.2%) suggesting possible cryptic and/or pseudocryptic diversification. Most of these possible cryptic and/or pseudocryptic species, as seen in Table 3, appear to be hidden within common species.

Three species, i.e. *Lebertiamaculosa, Monatractidesmadritensis* and *Torrenticolalaskai* appeared each with 2 BINs in our dataset. The intraspecific maximum distances between BINs within each of these species were below 3% (Suppl. material 1). On the other hand, the intraspecific maximum distances between BINs within each of the other eleven species in the dataset were greater than 5% (Suppl. material 1).

*Lebertiaglabra*, a species widely distributed in West Palaearctic (Di Sabatino et al. 2010) appeared in our dataset with 3 BINs. The first cluster (BIN:ACR9598) includes two specimens from Montenegro and The Netherlands; the second cluster (BIN:ACS0595) was more represented in BOLD and includes specimens from different parts of Europe - from The Netherlands and Poland to Montenegro, Italy and Macedonia. The third cluster (BOLD:AEI925) contained only specimens from Montenegro. The intraspecific K2P distances between all clusters ranged from 14.3 to 17.7% (Suppl. material 1).

*Lebertiainaequalis*, a species reported from the extended parts of the Palaearctic (Gerecke 2009, Di Sabatino et al. 2010), appeared in our dataset with 3 BINs, two of which each include only one specimen from Montenegro (BIN:AEF5913 and BIN:AEF2742, respectively). The third cluster (BIN:ADF6223), based on available records from BOLD, appears to be more widespread and contained specimens from The Netherlands, Poland and Montenegro. Intraspecific K2P distances between the latter cluster and BIN:AEF5913 was only 0.1%, while the distance from the second cluster (BIN:AEF2742) from Montenegro was rather large (17.3%; Suppl. material 1) highlighting the necessity of additional comprehensive morphological and molecu­lar analysis.

*Lebertiaporosa*, a eurytopic and eurythermous species, often reported from standing waters and pools of streams across the Holarctic (Gerecke 2009, Di Sabatino et al. 2010), is currently in the process of being revised (R. Gerecke, pers. communication) using DNA barcodes. Stur (2017) showed that 18 specimens of *L.porosa* from Norway comprise 7 BINs with a mean intraspecific *p*-distance of 11.7% and maximum up to 18.5%. In our dataset, specimens, morphologically assigned to *Lebertiaporosa*, were presented with two clusters. Based on the available records from BOLD, the first cluster (BIN:ACS0974) appeared to be well represented in the BOLD database with 133 records from different parts of Europe; the second cluster (BIN:AED4662) contained specimens only from Montenegro. In our study, specimens of the latter BIN were collected in large limnocrenic springs, such as Mareza and Vitoja, while specimens from the first cluster (BIN:ACS0974) were sampled in the lower reaches. The intraspecific K2P distance between these two *L.porosa* clusters in our dataset was estimated at 5.5% (Suppl. material 1).

*Sperchonbrevirostris*, a species inhabiting low-and middle order streams in the study area (Pešić et al. 2010, Pešić et al. 2018), was represented in our material by two clusters. Based on the available records from BOLD, the first cluster (BIN:ACP6107) includes specimens from Norway, Germany and one specimen from Montenegro, while the second cluster (BOLD:AED3857) contained three specimens from Montenegro and North Macedonia. The K2P distance between these two clusters was 8.1% (Suppl. material 1). Similarly, *S.clupeifer*, a species frequently reported from Western Palaearctic (Di Sabatino et al. 2010), appeared with two clusters in our dataset. The first cluster (BIN:ACS1100) is well represented in BOLD and includes specimens from different part of Europe, while the second cluster (BIN:AEE4061) contained a single specimen from Montenegro. The intraspecific K2P distance between these two clusters in our dataset was estimated at 8.3% (Suppl. material 1).

*Sperchonopsisverrucosa*, a species often reported from the Holarctic Region (Gerecke et al. 2016), was represented in our study with two clusters. The first cluster (BIN:ACS9705) was more represented in BOLD and includes specimens from Norway, Italy and one specimen from Montenegro. The second cluster (BIN:AEK8297) includes two specimens from Montenegro and Romania. The intraspecific K2P distances between these two clusters was 11.2%, indicating the need for additional integrative analysis.

*Torrenticolameridionalis*, a species originally described from Italy, is widely distributed in Montenegro, inhabiting mainly low order streams (Pešić et al. 2018). It is morphologically closely related to *T.elliptica* which remains distinguishable in the male sex only, based on the stouter genital field. In our COI tree (Suppl. material 2), *T.elliptica* appeared as a sister clade to the clade that includes two clusters morphologically assigned to *T.meridionalis* (BIN:AEI3402 and BOLD:AED7519, respectively). The intraspecific K2P distances between *T.elliptica* and *T.meridionalis* clusters ranged from 8.6-9.0%. On the other hand, the K2P distance between *T.meridionalis* clusters in our dataset was estimated at 6.6%.

*Atractidesnodipalpis*, a rhitrobiontic species, is the most frequently reported species of the genus in Europe (Gerecke et al. 2016). In our dataset, sequences of the specimens, morphologically assigned to the latter species, appeared as two clusters. Interestingly, specimens of both clusters were recorded syntopically. The first cluster (BIN:ACR0209) in the BOLD database was represented with 41 specimens from Norway (country of the type locality), The Netherlands, Montenegro and Russia, but also from Greenland. The second cluster (BIN:AED3547) includes two specimens from Montenegro. The intraspecific K2P distance between these two clusters was 18.8%, indicating the need for a comprehensive revision of this species complex.

*Hygrobatescalliger*, a rhitrobiontic species widely distributed in the Palaearctic (Di Sabatino et al. 2010), was represented by two clusters in our dataset, each with two records in the BOLD database. The first cluster (BIN:AEF4261) includes specimens from Norway and Montenegro, while the second cluster (BIN:AEL5782) includes specimens from Germany and Montenegro. The intraspecific K2P distance between these two clusters was 20.9% (Suppl. material 1), suggesting the existence of possible hidden cryptic and/or pseudocryptic species.

The sequences of *Atractidesgibberipalpis*, a rhitrobiontic species often reported from the Palaearctic (Pešić et al. 2021a), in our dataset were assigned to two different barcode clusters, each represented by a single specimen from Montenegro. The intraspecific K2P distance between these two clusters (BIN: BOLD:AEK7766 and BIN: BOLD:AEI3946, respectively) was estimated at 5% (Suppl. material 1).

*Unionicolaminor*, a species widely distributed in Europe (Gerecke et al. 2016), was presented with two clusters in our dataset. Based on available data from BOLD, the first cluster (BIN:AAU0335) includes specimens from Norway and The Netherlands and one specimen from Lake Šasko in Montenegro. The second cluster (BIN:AAU0335) includes specimens only from Montenegro. The intraspecific K2P distances between these two clusters in our dataset was 23.8% (Suppl. material 1), suggesting the existence of cryptic (or pseudocryptic, see Pešić and Smit (2016) for a discussion about pseudocryptic speciation in water mites) species. Stålstedt et al. (2013) showed that the Swedish population of *Unionicolaminor* consists of at least three cryptic species, emphasising the need for further research of the species in this complex.

Taxonomic studies of the above species were outside the scope of this paper. Further studies with material from a wider geographical area, were needed to clarify taxonomy and elucidate the delimitation of the species in the above complexes. This process should be accompanied by sufficient barcode coverage to allow the detection of phylogeographic patterns and/or even the existence of possible overlooked cryptic species. The build-up of DNA barcode library for water mites of Montenegro represents a long-term task, aimed at improving molecular identification and inclusion of this group in environmental assessment programmes and, on the other hand, to stimulate further biodiversity research of this limnofaunistic group in Montenegro and the Balkans.

## Supplementary Material

98C7E5CD-8838-52C3-9AAB-C45EA02AF488 10.3897/BDJ.9.e78311.suppl1 Supplementary material 1 Molecular distances Data type Molecular distances Brief description Molecular distances, based on the Kimura 2-parameter model of the analysed specimens of water mites from Montenegro. BINs are based on the barcode analysis from 15 November 2021. File: oo_613357.xlsx https://binary.pensoft.net/file/613357 Vladimir Pešić, Andrzej Zawal, Ana Manović, Aleksandra Bańkowska, Milica Jovanović

1AE967B4-F797-5CB6-A525-FD9034D049BC 10.3897/BDJ.9.e78311.suppl2 Supplementary material 2 Compact Neighbour-Joining tree Data type Neighbour-joining tree Brief description Compact Neighbour-Joining tree of all analysed water mite species based on Kimura 2-parameter distances. The tree was edited in MEGA7 (Kumar et al. 2016). Specimens are classified using ID numbers from BOLD and species name. BINs are based on the barcode analysis from 15 November 2021. Numbers next to nodes represent non-parametric boot­strap values (1,000 replicates, in %). The analyses involved all 233 COI nucleotide sequences. File: oo_613358.pdf https://binary.pensoft.net/file/613358 Vladimir Pešić, Andrzej Zawal, Ana Manović, Aleksandra Bańkowska, Milica Jovanović

## Figures and Tables

**Table 1.** Details on barcoded specimens from Montenegro.

**Taxa**	**Voucher Code**	**BOLD Process** **ID**	**BIN**	**Locality**	**Coordinates**
**Limnocharidae**					
*Limnocharesaquatica*	31. CG2020_6_C10	DNAEC032-20	BOLD:ACS0438	Podgorica, Zeta River at Pričelje	42.5022N, 19.2225E
**Hydryphantidae**					
*Panisusmichaeli*	CCDB 38361 A04	DCDDJ004-21	BOLD:ADT7504	Kolašin, Lalevića Dolovi, spring #1	42.899N, 19.631E
	CCDB 38361 A05	DCDDJ005-21		Kolašin, Lalevića Dolovi, spring #1	42.899N, 19.631E
	CCDB 38361 A06	DCDDJ006-21		Kolašin, Lalevića Dolovi, spring #1	42.899N, 19.631E
*Trichothyasjadrankae*	CCDB-38679-A08	DNCBD008-20	BOLD:AEF1286	Bar, Poseljanski stream at Poseljani	42.3095N, 19.0518E
*Partnunianaprintua*	CCDB 38361 A08	DCDDJ008-21	BOLD:AEL6734	Andrijevica, spring at Trešnjevik	42.7392N, 19.6933E
*Protziainvalvaris*	CCDB 38361 C11	DCDDJ035-21	BOLD:AEI2833	Kolašin, Bistrica stream	42.8054N, 19.4456E
	CCDB38233 A08	DCCDB008-21		Kolašin, Kolašinska rijeka stream	42.8391N, 19.5749E
	CCDB38233 A09	DCCDB009-21		Kolašin, Kolašinska rijeka stream	42.8391N, 19.5749E
	CCDB38233 A10	DCCDB010-21		Kolaši, Kolašinska rijeka stream	42.8391N, 19.5749E
*Protziasquamosapaucipora*	CCDB 38361 A09	DCDDJ009-21	BOLD:AEL1015	Kolašin, spring on road to Trešnjevik	42.7405N, 19.6801E
	CCDB 38361 A10	DCDDJ010-21		Kolašin, spring on road to Trešnjevik	42.7405N, 19.6801E
	CCDB 38361 A11	DCDDJ011-21		Kolašin, spring on road to Trešnjevik	42.7405N, 19.6801E
*Protziaoctopora*	CCDB38233 D09	DCCDB045-21	BOLD:AEI5747	Kolašin, Bistrica stream	42.9871N, 19.4338E
*Protziahalberti*	Hyd_MN_VP7	DNAEC081-20	BOLD:AED9646	Bijelo Polje, Lještanica stream	43.0631N, 19.5808E
	3. CG2020_8_2	DNAEC002-20		Bijelo Polje, Lještanica stream	43.0631N, 19.5808E
	4. CG2020_1	DNAEC003-20		Bijelo Polje, Lještanica stream	43.0631N, 19.5808E
	5. CG2020_1_3	DNAEC004-20		Bijelo Polje, Lještanica stream	43.0631N, 19.5808E
*Protziarotunda*	6. M18_01_1_D10	DNAEC045-20	BOLD:AED8976	Žabljak, Sedlo, spring Studenac	43.0973N, 19.0702E
	CCDB-3867-E04	DNCBD052-20		Bar, Međurječka rijeka stream	42.0363N, 19.2179E
	CCDB-3867-E05	DNCBD053-20		Bar, Međurječka rijeka stream	42.0363N, 19.2179E
*Protziarugosa*	6. CG2020_1_4	DNAEC005-20	BOLD:AEE010	Bijelo Polje, Lještanica stream	43.0631N, 19.5808E
	7. CG2020_8 B6	DNAEC017-20		Berane, spring nr Mon. Djurdjevi Stupovi	42.8527N, 19.862E
	CCDB38233 D05	DCCDB041-21		Mojkovac, Bistrica stream	42.9871N, 19.4338E
**Hydrodromidae**					
*Hydrodromareinhardi*	CCDB-3867-G04	DNCBD076-20	BOLD:AEF0798	Podgorica, Cijevna River at Dinoša	42.4057N, 19.3569E
*Hydrodromatorrenticola*	CCDB-3867-E06	DNCBD054-20	BOLD:AEF3799	Bar, Medjurječka rijeka stream	42.0363N, 19.2179E
**Lebertiidae**					
*Lebertiajadrensis*	CCDB 38361 C09	DCDDJ033-21	BOLD:ADK0383	Kolašin, Bistrica stream at Crkvine	42.8054N, 19.4456E
	CCDB-3867-G08	DNCBD080-20		Podgorica, Cijevna River at Dinoša	42.4057N, 19.3569E
	CCDB 38361 C08	DCDDJ032-21		Kolašin, Bistrica stream at Crkvine	42.8054N, 19.4456E
	CCDB-3867-F10	DNCBD070-20		Danilovgrad, spring below the bridge	42.5542N, 19.1059E
*Lebertiacuneifera*	CCDB 38363 A01	SEPTA001-21	BOLD:ADV4392	Nikšić, spring “Babino sicelo”	42.8043N, 19.2152E
*Lebertiavariolata*	CCDB-3867-B05	DNCBD017-20	BOLD:ADK0996	Bar, stream in Godinje Village	42.2206N, 19.1118E
	CCDB-3867-B07	DNCBD019-20		Bar, stream in Godinje Village	42.2206N, 19.1118E
	CCDB-3867-D03	DNCBD039-20		Bar, Rikavac stream above Old Bar	42.1001N, 19.1432E
	CCDB-3867-D04	DNCBD040-20		Bar, Rikavac stream above Old Bar	42.1001N, 19.1432E
	CCDB-3867-D05	DNCBD041-20		Bar, Rikavac stream above Old Bar	42.1001N, 19.1432E
	CCDB-3867-D06	DNCBD042-20		Bar, Rikavac stream above Old Bar	42.1001N, 19.1432E
	16. M19_24_3_E7	DNAEC054-20		Bar, Medjurječka rijeka stream	42.0226N, 19.22E
	17. M19_24_3_E8	DNAEC055-20		Bar, Medjurječka rijeka stream	42.0226N, 19.22E
*Lebertianatans*	CCDB38233 F03	DCCDB063-21	BOLD:AEF5684	Danilovgrad, spring below the bridge	42.5541N, 19.1057E
	CCDB38233 F04	DCCDB064-21		Danilovgrad, spring below the bridge	42.5541N, 19.1057E
	CCDB-3867-F06	DNCBD066-20		Danilovgrad, spring below the bridge	42.5542N, 19.1059E
*Lebertiaglabra*	CCDB38233 C04	DCCDB028-21	BOLD:AEI2925	Kolašin, Kolašinska rijeka stream	42.8391N, 19.5749E
	CCDB38233 D03	DCCDB039-21		Kolašin, Bistrica stream at Crkvine	42.9871N, 19.4338E
	CCDB38233 D04	DCCDB040-21		Kolašin, Bistrica stream at Crkvine	42.9871N, 19.4338E
	CCDB38233 D01	DCCDB037-21	BOLD:ACS0595	Mojkovac, Bistrica stream	42.9871N, 19.4338E
	CCDB38233 D02	DCCDB038-21		Mojkovac, Bistrica stream	42.9871N, 19.4338E
	CCDB38233 C05	DCCDB029-21	BOLD:ACR9598	Kolašin, Kolašinska rijeka stream	42.8391N, 19.5749E
*Lebertiainaequalis*	CCDB-3867-C03	DNCBD027-20	BOLD:AEF5913	Tuzi, Vitoja, pool	42.324N, 19.3637E
	CCDB-3867-B11	DNCBD023-20	BOLD:ADF6223	Tuzi, Vitoja, pool	42.324N, 19.3637E
	CCDB-3867-C02	DNCBD026-20		Tuzi, Vitoja, pool	42.324N, 19.3637E
	CCDB 38363 B04	SEPTA016-21		Bar, Skadar Lake at Murići	42.1637N, 19.2214E
	CCDB 38363 B06	SEPTA018-21		Bar, Skadar Lake at Murići	42.1637N, 19.2214E
	CCDB 38363 B10	SEPTA022-21		Podgorica, Skadar Lake at Donja Plavnica	42.2724N, 19.2007E
	CCDB 38363 B11	SEPTA023-21		Podgorica, Gornja Plavnica, river	42.2889N, 19.2108E
	CCDB-3867-E12	DNCBD060-20	BOLD:AEF2742	Bar, Medjurjecka rijeka stream	42.0363N, 19.2179E
*Lebertiainsignis*	CCDB38233 B12	DCCDB024-21	BOLD:AEB9107	Danilovgrad, River Zeta near Slap	42.6001N, 19.0656E
*Lebertiamaculosa*	32. CG2020_1_C11	DNAEC033-20	BOLD:AED9197	Bijelo Polje, Lještanica stream	43.0631N, 19.5809E
	33. CG2020_1_C12	DNAEC034-2		Bijelo Polje, Lještanica stream	43.0631N, 19.5809E
	1. CG2020_8	DNAEC001-20	BOLD:AED9718	Berane, spring nr. Mon. Djurdjevi Stupovi	42.8527N, 19.862E
	CCDB 38361 H01	DCDDJ085-21		Kolašin, spring at Monastir Morača	42.7668N, 19.3906E
*Lebertiaporosa*	CCDB-3867-G09	DNCBD081-20	BOLD:ACS0974	Podgorica, Cijevna River at Dinoša	42.4057N, 19.3569E
	CCDB 38363 C10	SEPTA034-21		Cetinje, River Crnojevića	42.3557N, 19.0228E
	CCDB38233 A01	DCCDB001-21	BOLD:AED4662	Podgorica, spring Mareza	42.4801N, 19.1822E
	7. CG2020_10	DNAEC006-20		Tuzi, Vitoja spring	42.3254N, 19.3628E
*Lebertiareticulata*	Hyd_MN_VP13	DNAEC086-20	BOLD:ADT9218	Šavnik, spring of Bukovica stream	43.0589N, 19.1103E
	Hyd_MN_VP14	DNAEC087-20		Šavnik, spring of Bukovica stream	43.0589N, 19.1103E
	CCDB 38363 A11	SEPTA011-21		Nikšić, spring Vukovo Vrelo	42.8574N, 18.9426E
*Lebertiaschechteli*	9. CG2020	DNAEC008-20	BOLD:AED9612	Žabljak, Sedlo, spring Studenac	43.0973N, 19.0702E
	10. CG2020_2_3	DNAEC009-20		Žabljak, Sedlo, spring Studenac	43.0973N, 19.0702E
**Oxidae**					
*Oxusangustipositus*	CCDB 38361 C03	DCDDJ027-21	BOLD:AEB9099	Ulcinj, Šasko Lake	41.9768N, 19.3388E
	CCDB-38679-A11	DNCBD011-20		Cetinje, Poseljanski stream, lower part	42.3057N, 19.0557E
	CCDB 38363 B05	SEPTA017-21		Bar, Skadar Lake at Murići	42.1637N, 19.2214E
	CCDB 38363 B07	SEPTA019-21		Bar, Skadar Lake at Murići	42.1637N, 19.2214E
**Teutoniidae**					
*Teutoniacometes*	33. M19_20_3_F11	DNAEC068-20	BOLD:ACH7884	Podgorica, Mareza canal	42.479N, 19.1813E
	Hyd_MN_VP5	DNAEC079-20		Danilovgrad, spring Svinjiška vrela	42.6384N, 19.0074E
**Sperchontidae**					
*Sperchonbrevirostris*	CCDB38233 D07	DCCDB043-21	BOLD:ACP6107	Mojkovac, Bistrica stream	42.9871N, 19.4338E
	CCDB38233 D08	DCCDB044-21	BOLD:AED3857	Mojkovac, Bistrica stream	42.9871N, 19.4338E
	CCDB38233 A11	DCCDB011-21		Kolašin, Kolašinska rijeka stream	42.8391N, 19.5749E
*Sperchonclupeifer*	Hyd_MN_VP11	DNAEC084-20	BOLD:AEE4061	Žabljak, Ljutica stream	43.1378N, 19.3023E
	CCDB-3867-B04	DNCBD016-20	BOLD:ACS1100	Bar, stream in Godinje Village	42.2206N, 19.1118E
*Sperchonhibernicus*	CCDB-3867-D02	DNCBD038-20	BOLD:AEF3824	Bar, Rikavac stream above Old Bar	42.1001N, 19.1432E
*Sperchonhispidus*	12. M19 29A 8_E3	DNAEC050-20	BOLD:AED3202	Danilovgrad, Zeta River at Spuž	42.5113N, 19.1982E
	29. CG2020_7_C8C7	DNAEC030-20		Danilovgrad, Zeta River at Spuž	42.5113N, 19.1982E
*Spechondenticulatus*	10. CG2020_8 B8	DNAEC019-20	BOLD:AED8428	Berane, spring nr. Mon. Djurdjevi Stupovi	42.8527N, 19.862E
*Sperchonpapillosus*	3. M19_12B_1_D7	DNAEC043-20	BOLD:AED2134	Budva, Lastva Grbaljska, stream	42.3103N, 18.8138E
*Sperchonthienemanni*	Hyd_MN_VP4	DNAEC078-20	BOLD:ADV4077	Šavnik, spring Kikov izvor near Boan	42.9465N, 19.1893E
	Hyd_MN_VP10	DNAEC083-20		Žabljak, Sedlo, Studenac spring	43.0972N, 19.0702E
	CCDB 38361 A03	DCDDJ003-21		Kolašin, Lalevića Dolovi, spring #1	42.899N, 19.631E
	CCDB 38363 A02	SEPTA002-21		Nikšić, Lukavica Mt., spring Babino Sicelo	42.8043N, 19.2152E
	CCDB 38363 A04	SEPTA004-21		Nikšić, Lukavica Mt., spring Babino Sicelo	42.8043N, 19.2152E
	CCDB 38363 A05	SEPTA005-21		Nikšić, Lukavica Mt., spring Babino Sicelo	42.8043N, 19.2152E
*Sperchonviolaceus*	Hyd_MN_VP8	DNAEC088-20	BOLD:AAN0076	Žabljak, Mlinski potok stream	43.1494N, 19.0898E
	27. M19_16A_3_F5	DNAEC062-		Kolašin, Biogradska River	42.8968N, 19.6047E
	56. CG2020_1	DNAEC010-20		Bijelo Polje, Lještanica stream	43.0631N, 19.5809E
	57. CG2020_8	DNAEC011-20		Bijelo Polje, Lještanica stream	43.0631N, 19.5809E
	58. CG2020	DNAEC012-20		Bijelo Polje, Lještanica stream	43.0631N, 19.5809E
	26. M19_16A_3_F4	DNAEC061-20		Kolašin, Biogradska River	42.8968N, 19.6047E
	28. M19_16A_3_F6	DNAEC063-20		Kolašin, Biogradska River	42.8968N, 19.6047E
	CCDB38233 D06	DCCDB042-21		Mojkovac, Bistrica stream	42.9871N, 19.4338E
	CCDB38233 H10	DCCDB094-21		Mojkovac, spring in Bistrica Village	42.9862N, 19.4349E
	CCDB38233 H11	DCCDB095-21		Mojkovac, spring in Bistrica Village	42.9862N, 19.4349E
*Sperchonopsisverrucosa*	CCDB 38361 B11	DCDDJ023-21	BOLD:AEK8297	Cetinje, spring “Smokov Vijenac”	42.254N, 18.9902E
	46. M19_16B_1_G10	DNAEC040-20	BOLD:ACS9705	Kolašin, Biogradska River	42.8968N, 19.6047E
**Torrenticolidae**					
*Monatractidesmadritensis*	CCDB-3867-G11	DNCBD083-20	BOLD:AED3803	Podgorica, Cijevna River at Dinoša	42.4057N, 19.3569E
	44. M19_12B_3_G8	DNAEC075-20		Budva, Lastva Grbaljska, first order stream	42.3103N, 18.8138E
	CCDB-3867-B01	DNCBD013-20	BOLD:AEL3852	Bar, stream in Godinje Village	42.2206N, 19.1118E
*Monatractidesstadleri*	CCDB38233 C03	DCCDB027-21	BOLD:AED3802	Bar, Rikavac stream above Old Bar	42.1001N, 19.1432E
	45. M19_129_3_G9	DNAEC076-20		Budva, Lastva Grbaljska, first order stream	42.3103N, 18.8138E
*Torrenticolaamplexa*	CCDB-3867-F08	DNCBD068-20	BOLD:ACR0665	Danilovgrad, spring below the bridge	42.5542N, 19.1059E
	CCDB-3867-F09	DNCBD069-20		Danilovgrad, spring below the bridge	42.5542N, 19.1059E
	CCDB38233 G04	DCCDB076-21		Danilovgrad, spring below the bridge	42.5542N, 19.1059E
*Torrenticolabrevirostris*	42. M19_29A_5_G6	DNAEC073-20	BOLD:AED9586	Danilovgrad, Zeta River at Spuž	42.5113N, 19.1982E
	CCDB 38363 C12	SEPTA036-21		Podgorica, Morača River in Podgorica	42.4368N, 19.2559E
*Torrenticoladudichi*	CCDB38233 D11	DCCDB047-21	BOLD:AED7520	Mojkovac, Bistrica stream	42.9871N, 19.4338E
	43. M19_16A_4_G7	DNAEC074-20		Kolašin, Biogradska rijeka stream	42.8968N, 19.6047E
*Torrenticolalaskai*	CCDB-3867-G06	DNCBD078-20	BOLD:AEF5471	Podgorica, Cijevna River at Dinoša	42.4057N, 19.3569E
	CCDB-3867-B10	DNCBD022-20		Kolašin, Tara River near Mateševo	42.7898N, 19.5374E
	CCDB-3867-E11	DNCBD059-20	BOLD:AED2306	Bar, Međurječka rijeka stream	42.0363N, 19.2179E
*Torrenticolalukai*	CCDB 38361 C12	DCDDJ036-21	BOLD:ACH9685	Kolašin, Bistrica stream at Crkvine	42.8054N, 19.4456E
*Torrenticolameridionalis*	CCDB 38361 D02	DCDDJ038-21	BOLD:AED7519	Kolašin, Bistrica stream at Crkvine	42.8054N, 19.4456E
	CCDB-3867-G02	DNCBD074-20		Bar, Orahovštica River	42.2476N, 19.0798E
	CCDB-3867-G01	DNCBD073-20		Bar, Orahovštica River	42.2476N, 19.0798E
	CCDB-3867-B09	DNCBD021-20		Kolašin, River Drcka near Mateševo	42.7619N, 19.5549E
	CCDB-3867-E01	DNCBD049-20		Bar, Rikavac stream above Old Bar	42.1001N, 19.1432E
	CCDB-3867-E03	DNCBD051-20		Bar, Rikavac stream above Old Bar	42.1001N, 19.1432E
	CCDB 38361 D01	DCDDJ037-21	BOLD:AEI3402	Kolašin, Bistrica stream at Crkvine	42.8054N, 19.4456E
	CCDB 38361 B08	DCDDJ020-21		Kolašin, Bistrica stream at Crkvine	42.8054N, 19.4456E
	CCDB38233 B10	DCCDB022-21		Kolašin, Kolašinska rijeka stream	42.8391N, 19.5749E
	CCDB38233 D12	DCCDB048-21		Mojkovac, Bistrica stream	42.9871N, 19.4338E
*Torrenticolasimilis*	CCDB 38361 B09	DCDDJ021-21	BOLD:AEK9661	Kolašin, Bistrica stream at Crkvine	42.8054N, 19.4456E
*Torrenticolabarsica*	CCDB-3867-E09	DNCBD057-20	BOLD:AEF1219	Bar, Međurječka rijeka stream	42.0363N, 19.2179E
	CCDB-3867-F04	DNCBD064-20		Bar, Međurječka rijeka stream	42.0363N, 19.2179E
*Torrenticolaelliptica*	CDB38233 B11	DCCDB023-21	BOLD:AEI9183	Kolašin, Kolašinska rijeka stream	42.8391N, 19.5749E
*Torrenticolaungeri*	19. M19_24_6_E10	DNAEC057-20	BOLD:AED2307	Bar, Međurječka rijeka stream	42.0226N, 19.22E
	20. M19_24_6_E11	DNAEC058-20		Bar, Međurječka rijeka stream	42.0226N, 19.22E
	CCDB-3867-D08	DNCBD044-20		Bar, Rikavac stream above Old Bar	42.1001N, 19.1432E
	CCDB-3867-G07	DNCBD079-20		Podgorica, Cijevna River at Dinoša	42.4057N, 19.3569E
*Pseudotorrenticolarhynchota*	CCDB-3867-B02	DNCBD014-20	BOLD:AEF1632	Bar, stream in Godinje Village	42.2206N, 19.1118E
	CCDB-3867-B03	DNCBD015-20		Bar, stream in Godinje Village	42.2206N, 19.1118E
**Limnesiidae**					
*Limnesiaundulata*	CCDB-3867-C05	DNCBD029-20	BOLD:AAX5286	Tuzi, Vitoja, pools	42.324N, 19.3637E
	CCDB 38363 C03	SEPTA027-21		Tuzi, Skadar Lake at Podhum	42.3139N, 19.3534E
**Hygrobatidae**					
*Atractidesfluviatilis*	CCDB-3867-G10	DNCBD082-20	BOLD:AEF1143	Podgorica, Cijevna River at Dinoša	42.4057N, 19.3569E
*Atractidesfissus*	CCDB38233 B03	DCCDB015-21	BOLD:AEI1811	Kolašin, Kolašinska rijeka stream	42.8391N, 19.5749E
	CCDB38233 D10	DCCDB046-21		Mojkovac, Bistrica stream	42.9871N, 19.4338E
*Atractidesanae*	1. CG2020_8 B3	DNAEC014-20	BOLD:AED1201	Berane, spring nr. Mon. Djurdjevi Stupovi	42.8527N, 19.862E
*Atractidesinflatipalpis*	29. M19_24_4_F7	DNAEC064-20	BOLD:AED3549	Bar, Međurječka rijeka stream	42.0226N, 19.22E
*Atractidesinflatipes*	CCDB-3867-G03	DNCBD075-20	BOLD:AEF1144	Bar, Orahovštica stream	42.2476N, 19.0798E
*Atractidesfonticolus*	CCDB38233 B09	DCCDB021-21	BOLD:AEI8720	Podgorica, Pričelje, spring Studenac	42.4835N, 19.2429E
	CCDB38233 B08	DCCDB020-21		Podgorica, Pričelje, spring Studenac	42.4835N, 19.2429E
*Atractidesgibberipalpis*	CCDB 38361 C07	DCDDJ031-21	BOLD:AEK7766	Mojkovac, Bistrica stream	42.8054N, 19.4456E
	CCDB38233 B02	DCCDB014-21	BOLD:AEI3946	Kolašin, Kolašinska rijeka stream	42.8391N, 19.5749E
*Atractidesinflatus*	14. M19_12_4_E5	DNAEC052-20	BOLD:ACB4677	Budva, Lastva Grbaljska, first order stream	42.3103N, 18.8138E
*Atractidesnodipalpis*	CCDB-3867-F07	DNCBD067-20	BOLD:ACR0209	Danilovgrad, spring below the bridge	42.5542N, 19.1059E
	41. M19_29A_1_G5	DNAEC072-20		Danilovgrad, Zeta River at Spuž	42.5113N, 19.1982E
	CCDB-3867-F05	DNCBD065-20	BOLD:AED3547	Danilovgrad, spring below the bridge	42.5542N, 19.1059E
	40. M19_29A_1_G4	DNAEC071-20		Danilovgrad, Zeta River at Spuž	42.5113N, 19.1982E
*Atractidespennatus*	CCDB-3867-F11	DNCBD071-20	BOLD:ADF7007	Bar, Orahovštica stream	42.2476N, 19.0798E
	CCDB-38679-A09	DNCBD009-20		Bar, Poseljani, Poseljanski stream	42.3057N, 19.0557E
	25. CG2020_9_C6	DNAEC028-20		Podgorica, Mareza spring	42.4801N, 19.1821E
	23. CG2020_9_C5	DNAEC027-20		Podgorica, Mareza spring	42.4801N, 19.1821E
	3. CG2020_2 B4	DNAEC015-20		Žabljak, Sedlo, Studenac spring	43.0973N, 19.0702E
	32. M19_23_1_F10	DNAEC067-20		Nikšić, Vidrovan, Vukovo Vrelo spring	42.8575N, 18.9414E
	31. M19_23_1_F9	DNAEC066-20		Nikšić, Vidrovan, Vukovo Vrelo spring	42.8575N, 18.9414E
	4. M19_22_1 D8	DNAEC042-20		Nikšić, spring in Miločani Village	42.8265N, 18.9018E
	CCDB 38363 C01	SEPTA025-21		Budva, spring Smokov Vijenac	42.2346N, 18.907E
	CCDB 38363 B12	SEPTA024-21		Budva, spring Smokov Vijenac	42.2346N, 18.907E
*Atractidesrobustus*	CCDB-3867-D12	DNCBD048-20	BOLD:ADZ9348	Bar, Rikavac stream above Old Bar	42.1001N, 19.1432E
	CCDB-3867-D11	DNCBD047-20		Bar, Rikavac stream above Old Bar	42.1001N, 19.1432E
	CCDB-3867-D10	DNCBD046-20		Bar, Rikavac stream above Old Bar	42.1001N, 19.1432E
	CCDB 38361 H02	DCDDJ086-21		Kolašin, spring nr. Monastir Morača	42.7668N, 19.3906E
	CCDB38233 B01	DCCDB013-21		Kolašin, Kolašinska Rijeka stream	42.8391N, 19.5749E
*Atractideslatipes*	18. M19_08B_7_E9	DNAEC056-20	BOLD:AED4000	Podgorica, River Cijevna at Trgaja	42.3964N, 19.3798E
*Atractidesstankovici*	CCDB38233 C08	DCCDB032-21	BOLD:AED3550	Dnilovgrad, River Zeta near Slap	42.6001N, 19.0656E
	CCDB38233 C07	DCCDB031-21		Danilovgrad, River Zeta near Slap	42.6001N, 19.0656E
	13. CG2020_4 B10	DNAEC020-20		Podgorica, Mareza canal	42.479N, 19.1813E
	14. CG2020_4 B11	DNAEC021-20		Podgorica, Mareza canal	42.479N, 19.1813E
*Hygrobatescalliger*	CCDB 38361 C06	DCDDJ030-21	BOLD:AEL5782	Kolašin, Crkvine, Bistrica stream	42.8054N, 19.4456E
	CCDB-38679-A04	DNCBD004-20	BOLD:AEF4261	Bar, Poseljanski stream at Poseljani	42.3095N, 19.0518E
	CCDB-38679-A03	DNCBD003-20		Bar, Poseljanski stream at Poseljani	42.3095N, 19.0518E
*Hygrobatesforeli*	Hyd_MN_VP6	DNAEC080-20	BOLD:AEE3281	Žabljak, Mlinski potok stream	43.1494N, 19.0898E
*Hygrobateslacrima*	27. CG2020_3_C7	DNAEC029-20	BOLD:AED2490	Kolašin, Tara River near Mateševo	42.7897N, 19.5383E
*Hygrobateslimnocrenicus*	13. M19_20_5_E4	DNAEC051-20	BOLD:AED2489	Podgorica, Mareza canal	42.479N, 19.1813E
*Hygrobateslongipalpis*	CCDB-3867-C07	DNCBD031-20	BOLD:ACR9783	Tuzi, Vitoja, pool	42.324N, 19.3637E
	CCDB-3867-C09	DNCBD033-20		Tuzi, Vitoja, pool	42.324N, 19.3637E
	CCDB-38679-A10	DNCBD010-20		Bar, Poseljani, Poseljanski stream	42.3057N, 19.0557E
	CCDB 38363 C04	SEPTA028-21		Tuzi, Skadar Lake at Podhum	42.3139N, 19.3534E
*Hygrobatesmediterraneus*	7. M19_24_2_D11	DNAEC046-20	BOLD:AED2190	Bar, Medjurječka rijeka stream	42.0226N, 19.22E
	8. M19_24_2_D12	DNAEC047-20		Bar, Medjurječka rijeka stream	42.0226N, 19.22E
	36. M19_24_1_G1	DNAEC070-20		Bar, Medjurječka rijeka stream	42.0226N, 19.22E
	CCDB-3867-F01	DNCBD061-20		Bar, Medjurječka rijeka stream	42.0363N, 19.2179E
*Hygrobatesnorvegicus*	Hyd_MN_VP3	DNAEC077-20	BOLD:ACH7323	Šavnik, spring Kikov izvor near Boan	42.9465N, 19.1893E
	CCDB 38361 A01	DCDDJ001-21		Kolašin, Lalevića Dolovi, spring #1	42.899N, 19.631E
	CCDB 38361 A02	DCDDJ002-21		Kolašin, Lalevića Dolovi, spring #1	42.899N, 19.631E
	CCDB 38361 A07	DCDDJ007-21		Kolašin, Lalevića Dolovi, spring #1	42.899N, 19.631E
**Unionicolidae**					
*Neumaniaimitata*	15. M19_29C_2_E6	DNAEC053-20	BOLD:AED4073	Danilovgrad, River Zeta at Spuž	42.5113N, 19.1982E
*Neumanialimosa*	CCDB-3867-C10	DNCBD034-20	BOLD:AEF5902	Tuzi, Vitoja, pool	42.324N, 19.3637E
	CCDB-3867-C01	DNCBD025-20		Tuzi, Vitoja, pool	42.324N, 19.3637E
	CCDB38233 G06	DCCDB078-21		Tuzi, Vitoja, pool	42.324N, 19.3637E
*Unionicolaminor*	CCDB-3867-G12	DNCBD084-20	BOLD:AEF4865	Ulcinj, Šasko Lake	41.9768N, 19.3389E
	CCDB 38361 C02	DCDDJ026-21		Ulcinj, Šasko Lake	41.9768N, 19.3389E
	CCDB 38361 C05	DCDDJ029-21		Ulcinj, Šasko Lake	41.9768N, 19.3389E
	CCDB 38363 B09	SEPTA021-21		Tuzi, Vitoja, pool	42.324N, 19.3637E
	CCDB 38361 C04	DCDDJ028-21	BOLD:AAU0335	Ulcinj, Šasko Lake	41.9768N, 19.3389E
*Unionicolaypsilophora*	CCDB 38363 D04	SEPTA040-21		Cetinje, River Cnojevica (*Anodontaexulcerata*)	42.3546N, 19.0286E
*Pionadamkoehleri*	CCDB 38361 B03	DCDDJ015-21	BOLD:AEK5107	Danilovgrad, Moromiš pond	42.5322N, 19.1993E
	CCDB 38361 B04	DCDDJ016-21		Danilovgrad, Moromiš pond	42.5322N, 19.1993E
	CCDB 38361 B05	DCDDJ017-21		Danilovgrad, Moromiš pond	42.5322N, 19.1993E
	CCDB 38361 B06	DCDDJ018-21		Danilovgrad, Moromiš pond	42.5322N, 19.1993E
	CCDB 38361 B07	DCDDJ019-21		Danilovgrad, Moromiš pond	42.5322N, 19.1993E
*Pionalaminata*	CCDB 38361 A12	DCDDJ012-21	BOLD:AEL3248	Danilovgrad, Moromiš pond	42.5322N, 19.1993E
*Pionadisparilis*	Hyd_MN_VP12	DNAEC085-20	BOLD:AEE3977	Šavnik, spring of Bukovica stream, pool	43.0589N, 19.1103E
	CCDB 38363 A08	SEPTA008-21		Nikšić, Vukovo Vrelo spring, pool	42.8577N, 18.9416E
	CCDB 38363 A09	SEPTA009-21		Nikšić, Vukovo Vrelo spring, pool	42.8577N, 18.9416E
*Typhistorris*	CCDB-3867-C08	DNCBD032-20	BOLD:AEF2208	Tuzi, Vitoja, pool	42.324N, 19.3637E
*Typhisornatus*	CCDB 38361 B01	DCDDJ013-21	BOLD:ACS0401	Danilovgrad, Moromiš pond	42.5322N, 19.1993E
	CCDB 38361 B02	DCDDJ014-21		Danilovgrad, Moromiš pond	42.5322N, 19.1993E
**Wettinidae**					
*Wettinalacustris*	30. M19_20_4_F8	DNAEC065-20	BOLD:ADL2726	Podgorica, Mareza canal	42.479N, 19.1813E
**Mideopsidae**					
*Mideopsismilankovici*	22. M19_24_2_E12	DNAEC059-20	BOLD:AED2191	Bar, Medjurječka rijeka stream	42.0226N, 19.22E
*Mideopsisroztoczensis*	CCDB-38679-A02	DNCBD002-20	BOLD:ACI1492	Cetinje, Poseljanski stream	42.3095N, 19.0518E
	CCDB-3867-G05	DNCBD077-20		Podgorica, Cijevna River at Dinoša	42.4057N, 19.3569E
	CCDB38233 C12	DCCDB036-21		Danilovgrad, Zeta River at Spuž	42.5112N, 19.1991E
	CCDB38233 C11	DCCDB035-21		Danilovgrad, Zeta River at Spuž	42.5112N, 19.1991E
	CCDB 38363 D07	SEPTA043-21		Danilovgrad, Zeta River at Vranjske Njive	42.4683N, 19.2579E
**Athienemanniidae**					
*Mundamellagermanica*	1. KIA_20B_D6	DNAEC041-20	BOLD:AED6269	Danilovgrad, Spuž, spring near Zeta River	42.5113N, 19.1982E
**Aturidae**					
*Hexaxonopsisserrata*	CCDB 38363 B01	SEPTA013-21		Bar, Skadar Lake at Murići	42.1637N, 19.2214E
	CCDB 38363 B02	SEPTA014-21		Bar, Skadar Lake at Murići	42.1637N, 19.2214E
	CCDB 38363 B03	SEPTA015-21		Bar, Skadar Lake at Murići	42.1637N, 19.2214E
*Parabrachypodamontii*	5. M19_20_6_D9	DNAEC044-20	BOLD:AED5455	Podgorica, Mareza canal	42.479N, 19.1813E
*Woolastokiarotundifrons*	10. M19_27_2_E1	DNAEC048-20	BOLD:AEE0289	Šavnik, Tušina River at Boan	42.9432N, 19.205E
	11. M19_27_2_E2	DNAEC049-20		Šavnik, Tušina River at Boan	42.9432N, 19.205E
**Arrenuridae**					
*Arrenuruscylindratus*	34. M19_20_1_F12	DNAEC069-20	BOLD:AED6864	Podgorica, Mareza canal	42.479N, 19.1813E
*Arrenurusrefractarioulus*	CCDB 38363 A07	SEPTA007-21		Nikšić, Lukavica Mt., pools	42.8118N, 19.1872E

**Table 2.** Summary table of K2P genetic distances within the different taxonomic levels derived from 233 analysed water mite specimens from Montenegro. The list of studied species is provided in Table 1. Deletion Method: Pairwise Deletion. Alignment: BOLD Aligner (Amino Acid based HMM).

**Label**	**n**	**Taxa**	**Comparisons**	**Min Dist. (%)**	**Mean Dist. (%)**	**Max Dist. (%)**	**SE Dist. (%)**
Within Species	200	53	391	0.00	2.43	24.16	0.01
Within Genus	207	14	2291	6.09	20.20	42.37	0.00
Within Family	168	7	1054	16.17	37.14	63.16	0.01

**Table 3.** Species with intraspecific (ISD) maximum pairwise distances > 2.2% (p-dist.). Divergence values were calculated for all studied sequences, us­ing the Nearest Neighbour Summary, implemented in the Barcode Gap Analysis tool provided by the Barcode of Life Data System (BOLD). BINs are based on the barcode analysis from 15 November 2021. Country codes (alpha-2 code): BG = Bulgaria, CH = Switzerland, DE = Germany, ES = Spain, FR = France, GB = United Kingdom, GL = Greenland, IT = Italy, NO = Norway, NL = Netherlands, ME = Montenegro, MK = North Macedonia, PL = Poland, RO = Romania, RS = Serbia, RU = Russia, SK = Slovakia. *n* = BIN member count.

**No.**	**Species**	**BIN**	***n***	**MeanISD**	**MaxISD**	**Country**	**Nearest BIN/Species**	**Distance to NN**
**1.**	*Lebertiaglabra*	BOLD:ACR9598	2	0.8	0.8	ME, NL	BOLD:ACS0595	12.52
	*Lebertiaglabra*	BOLD:ACS0595	20	0.64	1.36	NL, BG, ME, MK, IT, PL, SK	BOLD:AEJ3212	2.88
	*Lebertiaglabra*	BOLD:AEI925	3	0.64	0.96	ME	BOLD:ACO2179	12.02
**2.**	*Lebertiainaequalis*	BOLD:AEF5913	1	N/A	N/A	ME	BOLD:ADF6223	2.78
	*Lebertiainaequalis*	BOLD:ADF6223	18	0.18	0.34	NL, PL, ME	BOLD:AEF5913	2.78
	*Lebertiainaequalis*	BOLD:AEF2742	1	N/A	N/A	ME	BOLD:AEB4193	6.96
**3.**	*Lebertiamaculosa*	BOLD:AED9718	3	1.27	1.6	ME, MK	BOLD:AED9197	2.76
	*Lebertiamaculosa*	BOLD:AED9197	2	0.16	0.16	ME	BOLD:AED9718	2.76
**4.**	*Lebertiaporosa*	BOLD:ACS0974	133	0.81	2.37	NL, FR, ME, DE, GB, BG, IT, PL, SK, ES, CH	BOLD:AED4662	3.89
	*Lebertiaporosa*	BOLD:AED4662	12	0.14	0.85	ME	BOLD:ACS0974	3.89
**5.**	*Sperchonbrevirostris*	BOLD:AED3857	3	0.32	0.48	ME, MK	BOLD:AEK3053	2.72
	*Sperchonbrevirostris*	BOLD:ACP6107	28	0.55	3.12	NO, DE, ME	BOLD:AED3857	7.53
**6.**	*Sperchonclupeifer*	BOLD:ACS1100	11	1.68	3.47	NL, DE, NO, MK, ME, RU	BOLD:AEE4061	8.7
	*Sperchonclupeifer*	BOLD:AEE4061	1	N/A	N/A	ME	BOLD:ACS1100	8.7
**7.**	*Sperchonopsisverrucosa*	BOLD:AEK8297	1	N/A	N/A	ME, RO	BOLD:ACS0908	4.83
	*Sperchonopsisverrucosa*	BOLD:ACS9705	9	0.29	0.97	NO, IT, ME	BOLD:ADU8190	9.83
**8.**	*Monatractidesmadritensis*	BOLD:AED3803	2	0.16	0.16	ME	BOLD:AEL3852	1.44
	*Monatractidesmadritensis*	BOLD:AEL3852	2	0.64	0.64	ME, SR	BOLD:AED3803	1.44
**9.**	*Torrenticolameridionalis*	BOLD:AED7519	8	1.46	2.25	ME, MK	BOLD:AEI3402	6.57
	*Torrenticolameridionalis*	BOLD:AEI3402	4	1.42	2.09	ME	BOLD:AEK9662	6.25
**10.**	*Torrenticolalaskai*	BOLD:AEF5471	2	0.32	0.32	ME	BOLD:AED2306	2.17
	*Torrenticolalaskai*	BOLD:AED2306	4	0.82	1.34	RS, ME, RO	BOLD:AEF5471	2.17
11.	*Atractidesgibberipalpis*	BOLD:AEK7766	1	N/A	N/A	ME	BOLD:AEI3946	4.81
	*Atractidesgibberipalpis*	BOLD:AEI3946	1	N/A	N/A	ME	BOLD:AEK7766	4.81
**12.**	*Atractidesnodipalpis*	BOLD:ACR0209	41	0.59	3.05	NO, NL, GL, DE, ME, RS	BOLD:AED3548	13.3
	*Atractidesnodipalpis*	BOLD:AED3547	2	0	0	ME	BOLD:AAM4306	13.3
**13.**	*Hygrobatescalliger*	BOLD:AEF4261	2	1.2	1.2	NO, ME	BOLD:AEK4720	16.18
	*Hygrobatescalliger*	BOLD:AEL5782	2	1.03	1.03	DE, ME	BOLD:AEK4720	14.61
**14.**	*Unionicolaminor*	BOLD:AEF4865	3	0.59	0.7	ME	BOLD:ACI7165	17.02
	*Unionicolaminor*	BOLD:AAU0335	7	0.09	0.32	NO, NL, ME	BOLD:ACH3803	16.03
