# Supplementary figures and images for: A DNA barcode library for the water mites of Montenegro
Source: Biodivers Data J. 2021 Dec 20;9:e78311. doi: 10.3897/BDJ.9.e78311 (PMC8712502; doi:10.3897/BDJ.9.e78311)

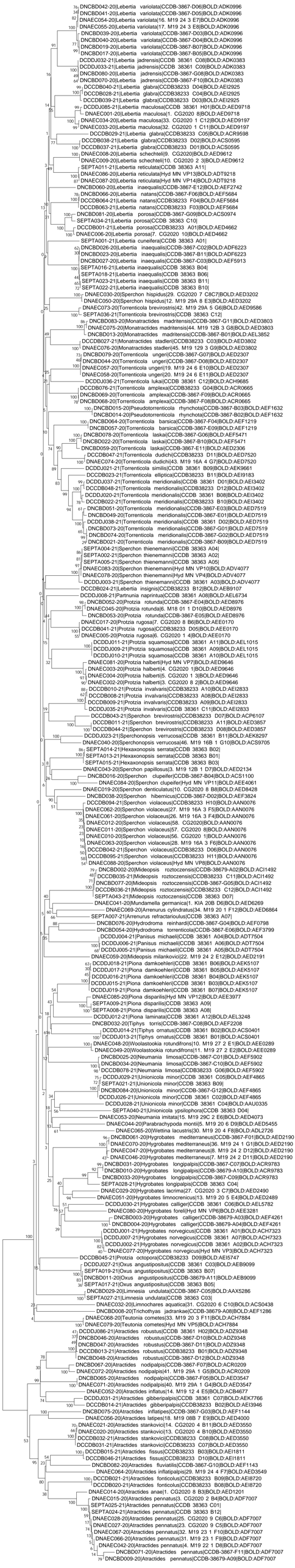

Supplement: Supplementary material 2 — Compact Neighbour-Joining tree [file bdj-09-e78311-s002.pdf]
